# The effects of acute exercise intensity on episodic and false memory among young adult college students

**DOI:** 10.15171/hpp.2019.20

**Published:** 2019-05-25

**Authors:** Emma K. Dilley, Liye Zou, Paul D. Loprinzi

**Affiliations:** ^1^Exercise & Memory Laboratory, Department of Health, Exercise Science and Recreation Management, The University of Mississippi, University, MS 38677, USA; ^2^Lifestyle (Mind-Body Movement) Research Center, College of Sport Science, Shenzhen University, Shenzhen 518060, China

**Keywords:** Aerobic exercise, Cognition, Fuzzy trace theory, Hippocampus, Memory distortion, Prefrontal cortex, Recognition, Recollection, Short-term memory

## Abstract

**Background:** Previous experimental work demonstrates that acute exercise may enhance episodic memory performance. However, limited research has examined the extent to which acute exercise influences false episodic memory production, and no studies, to date, have examined whether there is an intensity-specific effect of acute exercise on both true episodic and false episodic memories. Thus, the present experiment evaluated the effects of intensity-specific acute exercise on episodic memory and false episodic memory.

**Methods:** A three-arm, parallel, between-group randomized controlled trial was employed in the University setting, with participants (N=60; M_age_= 20.8 years) randomized into a moderate intensity exercise group (15-minute bout of treadmill exercise at 50% heart rate reserve), a high intensity exercise group (15-minute bout of treadmill exercise at 80% heart rate reserve), or a control group (time-matched period of sitting). True episodic and false episodic memory were both assessed using 6 word-lists from the Deese-Roediger-McDermott (DRM) paradigm, including both a short-term recall and a delayed memory recognition assessment.

**Results:** For the number of words recalled across each of the 6 lists, there was a significant main effect for list (P<0.001, η^2^_p_=0.15), marginally significant main effect for group (P=0.07, η^2^_p_=0.09), but no list by group interaction effect (P=0.44, η^2^_p_=0.03). Those in the high-intensity exercise group recalled significantly (P<0.05) more words than the control group. For the false episodic word recall, across various lists, high-intensity acute exercise was associated with a greater rate of false episodic memories. For the memory recognition task, there was no main effect for word type (P=0.46, η^2^_p_=0.01), group (P=0.4443, η^2^_p_=.03), word type by group interaction (P=0.44,η^2^_p_=0.03), recall by group interaction (P=0.4441, η^2^_p_=0.04), or word type by recall by group interaction (P=0.32, η^2^_p_=0.04). However, there was a main effect for recall (P<0.001, η^2^_p_=.54)and a word type by recall interaction (P<0.001, η^2^_p_=0.77).

**Conclusion:** These findings suggest that acute high-intensity exercise may enhance true episodic memories, and, possibly, also increase the rate of false episodic memories. We discuss these findings in the context of how different acute exercise intensities may have unique and differential effects on underlying mechanistic processes related to true and false episodic memory.

## Introduction

Emerging research demonstrates that acute exercise can help to facilitate episodic retrospective memory,^1^ but to date, few studies have examined the effects of exercise on false episodic memory function. False episodic memories can be conceptualized as a fabricated or distorted recollection of an event. Previous research has discussed potential mechanisms of false episodic memory.^2-5^ In brief, false episodic memories, or memory distortions, may arise from culturally determined expectations, labeling of the memory/event, and imperfect reality monitoring processes, such as source monitoring, which includes attributions about the origin of activated information. The source monitoring framework^6^ is perhaps one of the more extensive theoretical accounts of false episodic memories, which highlights several key aspects of false episodic memories.

These key aspects indicate that memory attributions arise from 1) various qualitative characteristics of the mental experience (e.g., perceptual, spatial, temporal, or emotional details), 2) the embeddedness of the mental experience (e.g., availability of supporting memories), and 3) goals, beliefs, motivation, and social factors. Per this model, false episodic memories occur because mental experiences arising from different events have overlapping characteristics that are imperfectly differentiated. Additional work also indicates that episodic memory and executive function performance predicts false episodic memory function.^4^ Both of these cognitive functions have been shown to be influenced by acute exercise,^7,8^ providing plausibility for a potential relationship between acute exercise and false episodic memory performance. Further, acute exercise may subserve the encoding of contextually specific information (reactivate verbatim memory traces and attenuate the reactivation of gist traces^9^), and in turn, minimize false episodic memory recall.

Lastly, as we have recently discussed,^10^ few studies have examined the potential intensity-specific effects of acute exercise on episodic memory, let alone false episodic memory function. Intensity-specific effects of acute exercise on memory are plausible; as discussed elsewhere,^10^ higher-intensity exercise may more favorably subserve long-term potentiation,^11^ and in turn, episodic memory performance. To address these two gaps in the literature, the purpose of this experiment was to evaluate the effects of acute exercise, across varying intensities, on true episodic memory and false episodic memory performance.

## Materials and Methods

### Study design

This experiment was approved by the authors’ institutional review board and participants provided written consent prior to participation. The present study was a three-arm, between-group randomized controlled trial, consisting of two exercise experimental groups and a control group. The senior author (PL) generated the randomization via a computer-facilitated algorithm. The lead researcher (ED) enrolled the participants and maintained allocation concealment by waiting to assign them to the group until after recruitment and consent was obtained. The exercise groups engaged in an acute 15-minute bout of moderate-intensity exercise or high-intensity exercise. The control group completed a time-matched seated task (on-line game). Both groups completed one laboratory visit in the authors’ Exercise & Memory Laboratory. Data collection occurred from September to December of 2018. Primary outcomes for this experiment included the true episodic memory and false episodic memory measures (described below). Secondary outcomes included physiological (e.g., heart rate) and psychological (e.g., ratings of perceived exercise) responses from the acute bout of exercise.

### Participants and procedures

All three groups included 20 participants (N=60). This is based from a power analysis indicating a sample size of 20 would be needed for sufficient power (d, 0.90; two-tailed α error probability, 0.05; 1-β error probability, 0.80). This was informed from other related work.^1,12,13^ We recruited through classroom announcements and word-of-mouth. Participants included male and females between the ages of 18 to 35 years. Additionally, participants were excluded if they: 1) Self-reported as a daily smoker^14,15^; 2) Self-reported being pregnant^16^; 3) Exercised within 5 hours of testing^1^; 4) Consumed caffeine within 3 hours of testing^17^; 5) Took medications used to regulate emotion (e.g., SSRI’s)^18^; 6) Had a concussion or head trauma within the past 30 days^19^; 7) Took marijuana or other illegal drugs within the past 30 days^20^; or 8) Were considered a daily alcohol user (> 30 drinks/month for women; > 60 drinks/month for men).^21^

### Experimental conditions

Similar to other related research,^22^ the control condition played a medium-level, on-line administered, Sudoku puzzle. Participants in this control group completed this time-matched puzzle for 20-minutes prior to completing the memory task (described below). The website for this puzzle is located here: https://www.websudoku.com/.

The two exercise conditions (moderate-intensity and vigorous-intensity) engaged in a 15-minute bout of treadmill exercise, followed by a 5-min recovery period. The HRR equation used to evaluate exercise intensity is:

HRR = [(HR_max_ - HR_rest_) * % intensity] + HR_rest_

To calculate HR_rest_, at the beginning of the visit, participants sat quietly for 5 minutes, and HR was recorded from a Polar HR monitor. HR_max_ was estimated from the 220-age formula. For the moderate-intensity and vigorous-intensity exercise, respectively, 50% and 80% will be entered into the above formula. These respective intensities represent moderate- and vigorous-intensity exercise.^23^

### Memory assessment

The procedure for this false episodic memory task was modeled after Roediger and McDermott.^24^ Participants listened (via headphones) to a recording of a list of 15 words; each word was read at a rate of 1 word per 1.5 seconds. They listened to six separate word lists. After each list, they were asked to write down (on paper) all the words they could remember from the list. As an example, each list was composed of associates (e.g., bed, rest, awake) of 1 non-presented word/lure (e.g., sleep). If, for example, they wrote down the word “sleep”, then this was evidence of constructing a false episodic memory. The lure word for list 1 was “mountain” and example studied words for this list were: hill, valley, and climb. The lure word for list 2 was “needle” and example studied words for this list were: thread, pin, eye. The lure word for list 3 was “chair” and example studied words for this list were: table, sit, and legs. The lure word for list 4 was “rough” and example studied words for this list were: smooth, bumpy, and road. The lure word for list 5 was “sleep” and example studied words for this list were: bed, rest, and awake. The lure word for list 6 was “sweet” and example studied words for this list were: sour, candy, and sugar.

After their recall of the 6th list, participants watched an on-line video (The Office Bloopers) for 10-minutes as a distractor task. After this, we assessed their false episodic memory recognition by giving them a piece of paper that has 42 words on it. Of these 42 words, 12 were words that they studied from one of the previous 6 lists. However, 30 were non-studied words. Among the 30 non-studied words, 6 were critical words/lures from which the lists were generated (e.g., sleep), 12 were unrelated to any of the items on the list, and 12 were related to the words on the lists (2 per list). The 42 items were subdivided into 6 blocks, with each block consisting of 7 items. Each block included 2 studied words, 2 related words, 2 unrelated words and the critical non-studied word/lure. For each of the 42 items, they were asked to rate the item on a 4-point scale, including the following response options: 4 for sure that they item was old (studied); 3 for probably old, 2 for probably new, and 1 for sure it was new.

### Statistical analysis

All statistical analyses were computed in JASP (v. 0.9.1). The proportion of items classified as Sure Old (a rating of 4), Probably Old (3), Probably New (2) and Sure New (1) were calculated. A 3 (group) x 4 (memory recognition categories) x 4 (word type; studied, unrelated lure, weakly related lure, or a critical lure) ANOVA was employed for the false episodic memory recognition assessment. For the number of words recalled, a 3 (group) x 6 (number of word lists) ANOVA was employed. Statistical significance was set at a nominal alpha of 0.05. Post hoc *t* tests were employed when main and interaction effects were statistically significant or approached statistical significance. Notably, we employed post-hoc tests when main or interaction effects also approached significance because relying on a specific threshold (e.g., 0.05) for statistical significance is problematic.^25,26^ Partial eta-squared (η^2^_p_) was calculated for effect size estimates.

## Results

Table 1 displays the demographic and behavioral characteristics of the sample. There were no significant differences in these parameters across the experimental groups. We also had no losses or exclusions after randomization.

Table 2 displays the physiological (heart rate) and psychological (rating of perceived exertion, RPE) responses to the experimental conditions. For both heart rate (*F*(6,171)=132.9, *P* < 0.001, η^2^_p_= 0.82) and RPE (*F*(6,171)=81.7, *P* < 0.001, η^2^_p_= 0.74), there was a significant time by group interaction. In the control group, heart rate remained in the upper 70’s and low 80’s bpm; in the moderate-intensity exercise group, heart rate increased from 81.7 bpm to 140 bpm (*P* < 0.001); and in the acute high-intensity exercise group, heart rate increased from 77.6 bpm to 170.2 bpm (*P* < 0.001).

**Figure 1:**
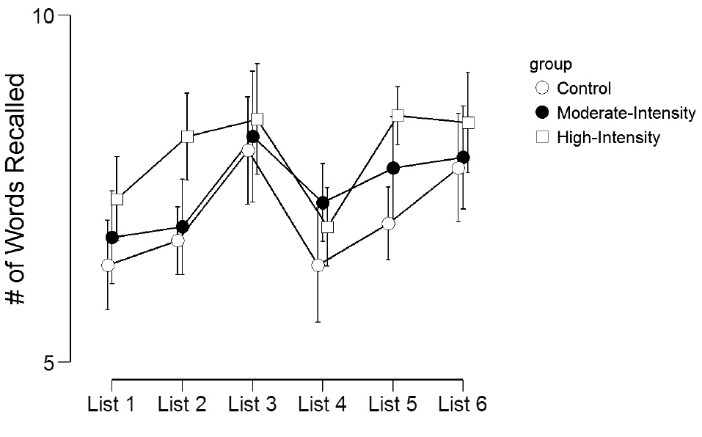
Number of words recalled across the 6 memory lists.

Table 3 and Figure 1 display the episodic memory recall scores across the experimental groups. For the number of words recalled across each list, there was a significant main effect for list (*F*(5,285)=10.2, *P* < 0.001, η^2^_p_= 0.15), marginally significant main effect for group (*F*(2,57)=2.7,* P* = 0.07, η^2^_p_= 0.09), but no list by group interaction effect (*F*(10,285)=1.00, *P* = 0.44, η^2^_p_= 0.03). Across the 6 lists, those in the high-intensity exercise group recalled significantly (*P* < 0.05) more words than the control group for List 1, List 2, and List 5. Similarly, the moderate-intensity exercise group recalled significantly more words than the control group for List 2.

**Figure 2:**
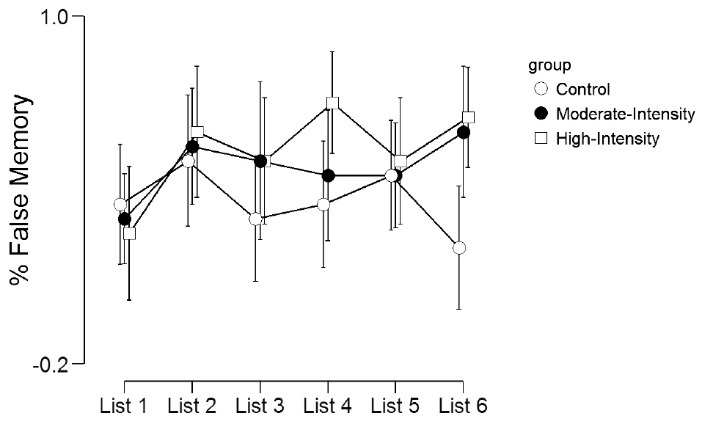
Proportion of false memories across the 6 lists.

The proportion of false episodic word recall is shown in Table 3 and Figure 2. There was no significant main effect for list (*F*(5,285)=2.15, *P* = 0.06, η^2^_p_= 0.04), group (*F*(2,57)=2.20, *P* = 0.12, η^2^_p_= 0.07) or list by group interaction (*F*(10,285)=1.27, *P* = 0.24, η^2^_p_= 0.04).

**Figure 3:**
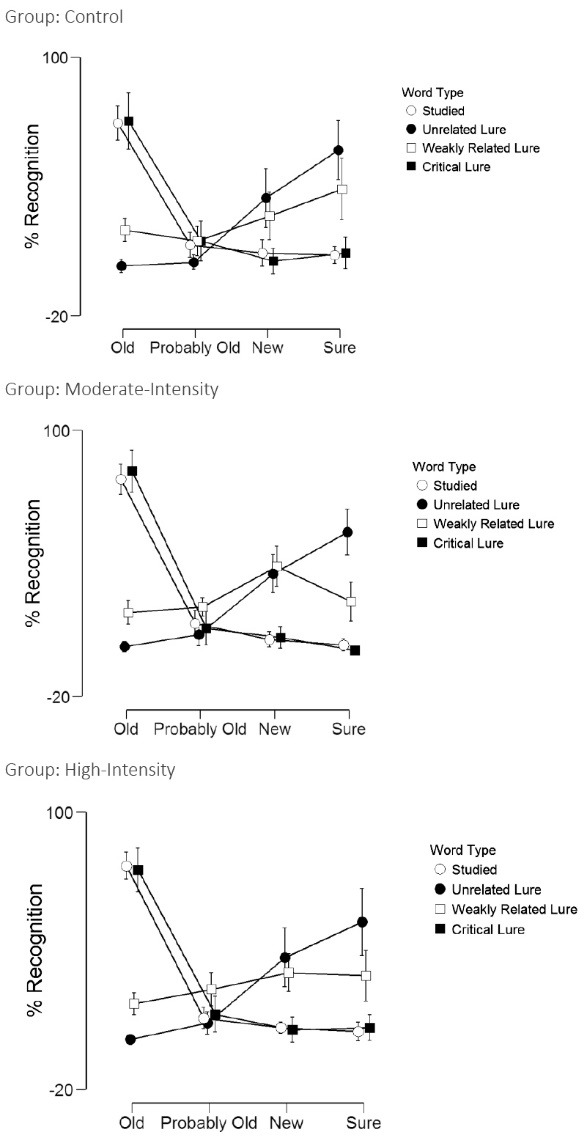
Recognition scores across word type and for each experimental condition.

Table 4 and Figure 3 display the memory recognition results. Word type refers to whether it was a studied, unrelated lure, weakly related lure, or a critical lure. Recall type refers to whether it was responded as sure old, probably old, probably new, or sure new. There was no main effect for word type (*F*(3,495) =0.85, *P* = 0.46, η^2^_p_= 0.01), group (*F*(2,55)=0.85,* P* = 0.43, η^2^_p_= 0.03), word type by group interaction (*F*(6,495)=0.97, *P* = 0.44, η^2^_p_= 0.03), recall by group interaction (*F*(6,495)=1.03, *P* = 0.41, η^2^_p_= 0.04), or word type by recall by group interaction (*F*(9,495)=1.13, *P* = 0.32, η^2^_p_= 0.04). However, there was a main effect for recall (*F*(3,495)=64.3, *P* < 0.001, η^2^_p_= 0.54) and a word type by recall interaction (*F*(9,495)=182.6, *P* < 0.001, η^2^_p_= 0.77). That is, participants across all three experimental conditions were more likely to perceive the studied and critical lure words as being “old” (i.e., that they previously were exposed to them during the study session).

## Discussion

This study evaluated the effects of acute exercise on true episodic memory and false episodic memory. In alignment with previous studies,^27,28^ with regard to acute exercise and episodic memory, the present experiment demonstrates that acute exercise improves episodic memory. Specifically, the acute high-intensity exercise group recalled significantly more words than the control group for three out of the 6 lists (e.g., Lists 1, 2, and 5). Similarly, the moderate-intensity exercise group recalled significantly more words than the control group for one out of the six lists (e.g., List 2). These results suggest that acute exercise is optimal for enhancing episodic memory and may occur in an intensity-specific fashion. This aligns with our other recent work suggesting that, for episodic memory, high-intensity exercise may be more beneficial than lower-intensity exercise.^10^ Regarding the false episodic word recall, there was no significant main effect for list, group, or list by group interaction, suggesting that exercise may have a less pronounced effect on false episodic memory recall, when compared to true episodic memory. The memory recognition results showed a significant main effect for recall and a word type by recall interaction. Participants from all three experimental conditions were more likely to perceive the studied and critical lure words as being “old” (i.e., that they previously were exposed to them during the study session).

Similar to the present experiment, our other work by Green et al^28^ and Siddiqui et al^27^ also modeled their false episodic memory task after Roediger and McDermott. The high false episodic memory rate was explained by exposure to the semantically related words. Green et al^28^ hypothesized that exposure to semantically related words may have caused activation of the related lure words, rendering participants to think that the words were previously stated during the encoding task. This explanation could be applied to the results that were found in the present study, as our current experiment also demonstrated a relatively high false episodic memory rate. Taken together, our employed false episodic memory paradigm was robust in inducing false episodic memories.

Limited research has examined the effects of exercise on false episodic memory recall. However, several of our past experimental studies have provided insight into the potential effects of acute exercise on false episodic memory. Siddiqui et al^27^ investigated the time course effects of acute exercise on false episodic memory. We demonstrated that acute exercise prior to the memory task may be optimal in enhancing episodic memory, which is the procedure that the present study utilized. Even though Siddiqui et al^27^ found no statistically significant results, for their false memory results the study presented evidence to suggest that acute exercise may reduce false episodic memory production. That is, both exercise conditions (before or during encoding) had lower false episodic memory scores when compared to the control condition. Siddiqui et al^27^ expounded their results with two suggestions. Firstly, that exercise prior to memory encoding may have a priming effect on the neurons, helping prepare them for integration into the memory trace, and overall, creating an optimal environment for true episodic memories.^11^ Secondly, that moderate-intensity exercise both before and during memory encoding may positively influence executive functioning, an important factor for reducing false episodic memories.^11^ Our follow-up work by Green et al^28^ explored the effects of acute exercise on prospective memory and false episodic memory. In the context of the false episodic memory recall results, there was some suggestive evidence that acute exercise reduced the production of false episodic memories.

Although the present experiment did not provide strong evidence of a consistent relationship between exercise intensity and false episodic memory recall, our findings provide some suggestive evidence that higher-intensity exercise may actually increase the likelihood of false episodic memories. This is in contrast to the findings of Green et al^28^ and Siddiqui et al^27^ that employed moderate-intensity exercise protocols. These potential intensity-dependent effects may be a result of the effect that acute exercise intensity has on true episodic memory. Higher-intensity acute exercise is more effective in enhancing true episodic memory, and given that our employed false episodic memory paradigm (Roediger and McDermott) involves a critical lure word that is highly semantically related to the studied words, it is plausible that higher-intensity exercise may actually increase the likelihood of false episodic memories, when compared to lower-intensity exercise (such as moderate-intensity exercise). Per the fuzzy trace theory,^29^ when a memory is encoded, two memory traces are formed, including a verbatim trace and a gist trace, with the latter more likely to decay over time. Speculatively, higher-intensity exercise may be more effective in stabilizing both traces, given the role of higher-intensity exercise on synaptic plasticity. Further, given the role of the prefrontal cortex in inhibiting false episodic memories,^30^ it is possible that moderate-intensity exercise, which activates the prefrontal cortex,^31^ may help reduce false episodic memories, whereas high-intensity exercise, which reduces prefrontal cortex activity,^32^ may accentuate false episodic memories. This is in support of findings form another laboratory that showed that chronic training, in which aerobic fitness was enhanced, increased the ability to correctly identify lure stimuli as similar.^33^ These assertions align with the accumulating body of research on this topic, including our past two studies by Green et al and Siddiqui et al, along with the present study’s findings.

In addition to false episodic memory, future work should also continue to evaluate whether there is an intensity-specific effect of acute exercise on true episodic memory. As stated, our present findings suggest that higher-intensity exercise is more beneficial for enhancing true episodic memories. This finding aligns with the conclusions of our recent systematic review on this topic.^10^ However, as noted in our systematic review, very few studies have directly compared different exercise intensities within the same study. The present experiment bridges this gap in the literature by directly comparing control, moderate-intensity and high-intensity exercise protocols. We have discussed these intensity-specific mechanisms in detail elsewhere,^7,34^ which, in brief, include intensity-specific effects on long-term potentiation. Future work should continue to evaluate whether there is a potential intensity-specific effect of acute exercise on true episodic and false episodic memories.

In conclusion, this study examined the intensity-specific effects of acute exercise on episodic memory and false episodic memory. We did not observe a strong, consistent association between acute exercise and false episodic word recall; however, we did observe evidence to suggest that acute exercise, particularly high-intensity exercise, can improve true episodic memory. Future work on this novel line of inquiry should aim to overcome the limitations of our present experiment. For example, such work should employ a more heterogeneous, representative sample, as well as utilize a within-subject design.

## Ethical approval

This study was approved by the University of Mississippi’s ethics committee (#19-003).

## Competing interests

The authors declare that they have no competing interests.

## Funding

None.

## Authors’ contributions

ED was involved in study conceptualization, data collection and manuscript writing; LZ was involved in manuscript revising; and PL was involved in study conceptualization, statistical analyses and manuscript writing.

**Table 1:** Demographic and behavioral characteristics of the sample

**Variable**	**Control (n = 20)**	**Moderate-Intensity (n** **= 20)**	**High-Intensity (** ***N *** **= 20)**	***P*** ** value**
Age, mean years	20.5 (1.1)	20.8 (1.1)	21.1 (0.9)	0.31
Gender, % female	80.0	95.0	95.0	0.19
Race-Ethnicity, % White	70.0	70.0	80.0	0.88
BMI, mean kg/m^2^	27.5 (6.2)	25.0 (5.8)	25.7 (6.2)	0.42
MVPA, mean min/wk	131.9 (116.1)	103.3 (91.1)	153.4 (106.5)	0.33
Affect, mean				
Positive	28.2 (7.0)	29.0 (7.2)	27.5 (7.9)	0.80
Negative	12.3 (2.4)	13.4 (3.6)	13.0 (2.7)	0.50

BMI, body mass index; MVPA, moderate-to-vigorous physical activity. Values in parentheses are standard deviations; *P* value is calculated from a one-way ANOVA (continuous variables) or chi-square analysis (categorical variables).

**Table 2:** Physiological (heart rate) and psychological (rating of perceived exertion) responses

**Variable**	**Control (n** **= 20)**	**Moderate-Intensity (n** **= 20)**	**High-Intensity (n** **= 20)**	***P*** ** value**
Heart rate, mean bpm				
Rest	79.9 (12.6)	81.7 (11.7)	77.6 (18.3)	*F*(3,171; time)=466.9, *P *< 0.001, η^2^_p_=0.89 *F*(2,57; group)=81.6, *P *< 0.001, η^2^_p_=0.74 *F*(6,171; time x group)=132.9, *P *< 0.001, η^2^_p_=0.82
Midpoint	80.8 (11.7)	138.8 (12.7)	149.9 (19.6)	
Endpoint	79.8 (11.2)	140.1 (7.2)	170.2 (13.1)	
5-Minutes post	82.2 (10.9)	91.2 (13.8)	97.0 (14.3)	
RPE, mean				
Rest	6.3 (0.9)	6.0 (0.0)	6.0 (0.0)	*F*(3,171; time)=320.9, *P *< 0.001, η^2^_p_=0.85 *F*(2,57; group)=35.4, *P *< 0.001, η^2^_p_=0.55 *F*(6,171; time x group)=81.7, *P *< 0.001, η^2^_p_=0.74
Midpoint	6.6 (1.1)	11.1 (1.4)	11.4 (1.7)	
Endpoint	6.9 (2.4)	11.4 (1.3)	14.3 (1.6)	
5-Minutes post	6.9 (2.2)	6.5 (0.8)	7.1 (1.1)	

**Table 3:** True and false episodic memory recall scores across the three groups

**Variable**	**Control (n** **= 20)**	**Moderate-Intensity (n** **= 20)**	**High-Intensity (n** **= 20)**	***P*** ** value**
**True episodic word recall**				F(5,285; list)=10.2, *P*<0.001, η^2^_p_=0.15 F(2,57; group)=2.7, *P*=0.07, η^2^_p_=0.09 F(10,285; list x group)=1.00, *P*=0.44, η^2^_p_=0.03
List 1, mean # words	6.4 (1.2)	6.8 (1.4)	7.4 (1.7)^a^	
List 2, mean # words	6.7 (1.2)	6.9 (1.7)^c^	8.2 (1.6)^b^	
List 3, mean # words	8.0 (1.9)	8.2 (2.7)	8.5 (1.6)	
List 4, mean # words	6.4 (2.0)	7.3 (2.1)	6.9 (1.4)	
List 5, mean # words	7.0 (1.5)	7.8 (2.2)	8.6 (1.7)^b^	
List 6, mean # words	7.8 (2.0)	7.9 (1.7)	8.5 (2.2)	
**False episodic word recall**				F(5,285; list)=2.15, *P*=0.06, η^2^_p_=0.04 F(2,57; group)=2.20, *P*=0.12, η^2^_p_=0.07 F(10,285; list x group)=1.27, *P*=0.24, η^2^_p_=.04
List 1, % false recall	35.0 (48.9)	30.0 (47.0)	25.0 (44.4)	
List 2, % false recall	50.0 (51.3)	55.0 (51.0)	60.0 (50.3)	
List 3, % false recall	30.0 (47.0)	50.0 (51.3)	50.0 (51.3)	
List 4, % false recall	35.0 (48.9)	45.0 (51.0)	70.0 (47.0)^a^	
List 5, % false recall	45.0 (51.0)	45.0 (51.0)	50.0 (51.3)	
List 6, % false recall	20.0 (41.0)	60.0 (50.3)^c^	65.0 (48.9)^b^	

^a^ High-intensity different (*P *< 0.05) than control. ^b^ High-intensity different (*P *< 0.01) than control. ^c^ Moderate-intensity different (*P *< 0.05) than control.

**Table 4:** Memory recognition scores for each word type across the three groups (N=60)

**Word Type**	**Recall**	**Group**	**Mean**	**SD**
Studied	Sure Old	Control	69.456	15.403
		Moderate-Intensity	77.920	14.113
		High-Intensity	76.700	12.267
	Probably Old	Control	12.956	11.163
		Moderate-Intensity	12.915	11.930
		High-Intensity	10.820	9.405
	Probably New	Control	9.261	12.086
		Moderate-Intensity	5.835	7.215
		High-Intensity	6.735	5.027
	Sure New	Control	8.328	7.571
		Moderate-Intensity	3.330	5.673
		High-Intensity	4.995	8.284
Unrelated Lure	Sure Old	Control	3.239	5.819
		Moderate-Intensity	2.495	4.757
		High-Intensity	1.665	3.417
	Probably Old	Control	5.089	6.485
		Moderate-Intensity	7.920	10.289
		High-Intensity	8.750	9.928
	Probably New	Control	34.722	26.546
		Moderate-Intensity	35.410	17.502
		High-Intensity	37.085	26.423
	Sure New	Control	56.944	26.555
		Moderate-Intensity	54.170	21.552
		High-Intensity	52.500	29.878
Weakly Related Lure	Sure Old	Control	19.917	10.366
		Moderate-Intensity	17.905	10.905
		High-Intensity	17.075	9.922
	Probably Old	Control	14.806	13.266
		Moderate-Intensity	20.430	8.334
		High-Intensity	23.330	14.700
	Probably New	Control	26.394	21.252
		Moderate-Intensity	38.715	18.635
		High-Intensity	30.420	16.933
	Sure New	Control	38.883	27.421
		Moderate-Intensity	22.920	18.109
		High-Intensity	29.170	22.541
Critical Lure	Sure Old	Control	70.361	25.276
		Moderate-Intensity	81.650	19.426
		High-Intensity	74.985	19.870
	Probably Old	Control	14.778	17.957
		Moderate-Intensity	10.835	15.550
		High-Intensity	12.510	16.110
	Probably New	Control	5.556	11.428
		Moderate-Intensity	6.675	9.977
		High-Intensity	5.835	11.177
	Sure New	Control	9.267	14.262
		Moderate-Intensity	0.835	3.734
		High-Intensity	6.670	11.341
